# Exciton–Exciton Interactions in Coaxial Double Quantum Rings

**DOI:** 10.3390/nano9101469

**Published:** 2019-10-16

**Authors:** Vanik Shahnazaryan, Vram Mughnetsyan, Ivan Shelykh, Hayk Sarkisyan

**Affiliations:** 1 Faculty of Physics and Engineering, ITMO University, 197101 St. Petersburg, Russia; ivshel@mail.ru; 2 Faculty of Physics, Yerevan State University, Yerevan 0025, Armenia; vram@ysu.am (V.M.); shayk@ysu.am (H.S.); 3 Science Institute, University of Iceland, IS-107 Reykjavik, Iceland; 4 Institute of Engineering and Physics, Russian–Armenian (Slavonic) University, Yerevan 0026, Armenia

**Keywords:** quantum ring, exciton, Coulomb interactions

## Abstract

We study theoretically the quantum states of two interacting excitons in coaxial double quantum rings. An interplay between exciton–exciton Coulomb interactions and specific geometry of the structure leads to the emergence of peculiar energy spectrum of two exciton system. We develop a semi-analytic approach providing highly accurate energies of system in the wide range of values of geometrical parameters relevant to experimental realizations.

## 1. Introduction

Rapid development of nanolitography and epitaxial techniques made possible fabrication of semiconductor heterostructures of complex geometry. Among them, quantum rings are of particular interest [1,2]. Being non-simply connected structures, mesoscopic rings demonstrate a set of fundamental purely quantum phenomena related to the phase of a wavefunction, such as Aharonov–Bohm [3], Altshuler–Aronov–Spivak [4] and Aharonov–Casher [5] oscillations. Investigations in this field revealed further the quantum nature of single- and many-electron states in the rings including the demonstration of the cumulative impact of impurities and magnetic field [6,7], the onset of persistent currents [8,9], the analysis of the possibilities of spintronic applications [10,11], the study of rotational and vibrational spectra of few electron states [12], Kondo [13] and geometric effects [14,15], impurities [16], electron–electron interactions [17] and magnetic field impact on few electron states [18]. More complicated geometries, such as double coaxial quantum rings [19], were also studied. The recent advances in this area included the calculation of the absorption coefficients of single electron states for various configurations of the system [20,21,22,23], model [24,25] and first-principle [26] analysis of few-electron states, and investigation of the possibility of photon antibunching [27].

Quantum rings also provide a platform for optoelectronic applications related to their excitonic properties. Extensive studies of excitons in quantum rings started immediately after their observation [28]. Such phenomena as excitonic analogs of Aharonov–Bohm effect [29,30,31,32,33,34,35,36,37], excitonic persistent currents induced by circular polarized light [38], optical magneto-oscillations [39] and strong exciton–photon coupling [40] were investigated. Excitonic properties of double coaxial rings also received some attention [41,42,43].

Among the most fascinating properties of excitons in various mesoscopic structures is the possibility to obtain pronounced optical nonlinearities, stemming from the interparticle scattering. Exciton–exciton interactions were analyzed in detail in quantum wells [44,45,46,47], double quantum wells [48], transition-metal dichalcogenide monolayers [49,50] and quantum wires [51]. In the domain of physics of quantum rings, the impact of Coulomb interactions on Aharonov–Bohm effect was studied for few electron configurations both theoretically [24] and experimentally [52,53]. In addition, the combined effect of electron–hole interaction and the radial confinement of particles on the exciton energy spectra was studied [54,55]. In the current paper, we study the peculiarities of the Coulomb interaction processes of excitons localized in double coaxial ring structures. The rotational symmetry of considered structure determines the cyclic nature of scattering processes. In addition, in contrast to the case of quantum well excitons, the structural peculiarities of the system prevent the full separation of center of mass and relative dynamics of double exciton system. We found that these circumstances have a decisive impact on the dynamics of two excitons, leading to a formation of specific energetic spectrum.

The paper is organized as follows. In Section 2, we analyze single exciton states in an individual quantum ring. In Section 3, we analyze the properties of exciton–exciton interactions and present the emerging energy spectrum of the system. Conclusions summarize the obtained results.

## 2. Exciton States in an Individual Quantum Ring

**Figure 1:**
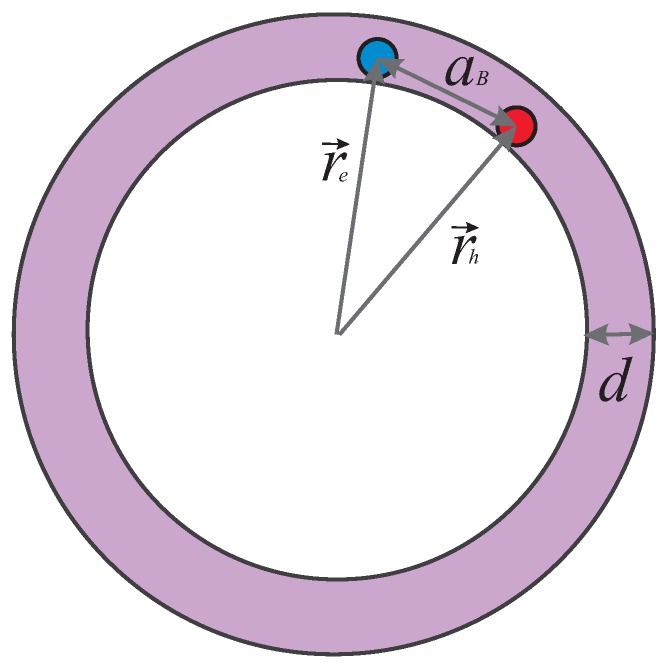
The sketch of an exciton localized in a single quantum ring of the width *d*. In the case of a narrow ring $d\ll |{\vec{r}}_{e,h}|$, the exciton state can be treated as quasi one-dimensional. Here, $a_{B}$ denotes Bohr radius of an exciton.

Let us consider an exciton inside an individual quantum ring, as shown in Figure 1. The Hamiltonian of an electron–hole pair confined in this geometry has the following form:

(1) $$\begin{array}{c} {\hat{H}}_{exc}=-\frac{\hbar^{2}}{2m_{e}}\Delta_{e}+V_{\mathrm{conf}}\left(r_{e}\right)-\frac{\hbar^{2}}{2m_{h}}\Delta_{h}+V_{\mathrm{conf}}\left(r_{h}\right)-\frac{\alpha_{C}}{|{\vec{r}}_{e}-{\vec{r}}_{h}|}, \end{array}$$

where the first (second) two terms describe the kinetic motion and confinement potential in the radial direction for the electron (hole). The last term in the Hamiltonian corresponds to the Coulomb interaction between an electron and a hole, with $\alpha_{C}=e^{2}/\left(4\pi \varepsilon_{0}\varepsilon \right)$, and ε denotes the dielectric permittivity of the media. In the current paper, we restrict the discussion to the situation of the strong confinement of the particles in the radial direction, which agrees well with state-of-the-art experimental realizations of quantum rings [42]. This means that electron–hole interaction does not change sufficiently the radial part of the wavefunction, and one can seek the solution of the stationary Schrodinger equation with the Hamiltonian (Equation (1)) in the form:

(2) $$\Psi \left({\vec{r}}_{e},{\vec{r}}_{h}\right)=f\left(r_{e}\right)f\left(r_{h}\right)\psi \left(\varphi_{e},\varphi_{h};R\right),$$

where $f(r_{e[h]})$ is the ground state solution of the single particle radial Schrodinger equation

(3) $$\begin{array}{c} -\frac{\hbar^{2}}{2m_{e[h]}}\left[\frac{d^{2}}{dr_{e[h]}^{2}}+\frac{1}{r_{e[h]}}\frac{d}{dr_{e[h]}}\right]f\left(r_{e[h]}\right)+V_{\mathrm{conf}}\left(r_{e}\left[h\right]\right)f\left(r_{e[h]}\right)=E_{e[h]}^{\mathrm{rad}}f\left(r_{e[h]}\right), \end{array}$$

$\varphi_{e}$, $\varphi_{h}$ are the angular coordinates of electron and hole, and *R* is the effective radius of the ring.

The last factor in Equation (2) is the eigenfunction of the angular Coulomb problem with the effective interaction potential given by:

(4) $$\begin{array}{c} V_{\mathrm{eff}}\left(\varphi_{e},\varphi_{h}\right)=-\alpha_{C}\int \frac{f^{2}\left(r_{e}\right)f^{2}\left(r_{h}\right)}{|{\vec{r}}_{e}-{\vec{r}}_{h}|}r_{e}r_{h}dr_{e}dr_{h}=-\alpha_{C}\int \frac{f^{2}\left(r_{e}\right)f^{2}\left(r_{h}\right)}{\sqrt{r_{e}^{2}+r_{h}^{2}-2r_{e}r_{h}\cos\left(\varphi_{e}-\varphi_{h}\right)}}r_{e}r_{h}dr_{e}dr_{h}. \end{array}$$

The validity of the adiabatic ansatz in Equation (2) stems from the evidence that the energies of radial $E_{e}^{\mathrm{rad}}$ and angular $E_{e}^{\mathrm{ang}}$ quantization of particle motion scale as $d^{-2}$ and $R^{-2}$, respectively, where *d* is the thickness of the ring. Hence, in the limit of narrow rings, with typical values d=1.5 nm and R=25 nm, one has $E_{e}^{\mathrm{ang}}/E_{e}^{\mathrm{rad}}\approx 0.003$.

To proceed further, we introduce the center of mass $\varphi =(m_{e}\varphi_{e}+m_{h}\varphi_{h})/M$ and relative $\vartheta =\varphi_{e}-\varphi_{h}$ angular coordinates, with $M=m_{e}+m_{h}$ and $\mu ={\left(m_{e}^{-1}+m_{h}^{-1}\right)}^{-1}$ being exciton total and reduced masses, respectively. We can then treat the dynamics of the center of mass and relative angular motion separately, factorizing the wavefunction as:

(5) $$\psi_{J}\left(\varphi ,\vartheta ;R\right)=\frac{1}{\sqrt{2\pi }}\exp\left(iJ\varphi \right)\chi \left(\vartheta ,R\right),$$

where J=0,±1,…. We further note that given the rotational symmetry of the problem Coulomb interaction is of the periodic character: $V_{\mathrm{eff}}\left(\vartheta ;R\right)=V_{\mathrm{eff}}\left(\vartheta +2\pi i,R\right)$, i=0,±1,…. To account this periodicity, one can represent the wavefunction of the relative motion χ(ϑ,R) using the following tight-binding ansatz [29,32]:

(6) $$\chi \left(\vartheta ,R\right)=\frac{1}{\sqrt{N}}\sum_{i}U\left(R\vartheta_{i}\right),$$

where $\vartheta_{i}=\vartheta +2\pi i$, *N* is normalization constant and $U(R\vartheta_{i})$ is the eigenfunction of Shcrodinger equation

(7) $$\left(-\frac{\hbar^{2}}{2\mu R^{2}}\frac{d^{2}}{d\vartheta_{i}^{2}}-\frac{\alpha_{C}}{|R\vartheta_{i}|+\gamma d}\right)U\left(R\vartheta_{i}\right)=E_{0}U\left(R\vartheta_{i}\right),$$

with γ being a cut off scaling parameter. We approximated Coulomb interaction $V_{\mathrm{eff}}\left(\vartheta ;R\right)$ by Loudon model potential [56,57,58], previously applied for the description of the excitonic states in carbon nanotubes [59,60] and semiconductor quantum wires [61,62,63]. The solution of Equation (7) can be written in terms of Whittaker’s function:

(8) $$U_{\alpha }\left(R\vartheta_{i}\right)=CW_{\alpha ,1/2}\left[2(|R\vartheta_{i}\left|+\gamma d\right)/\left(\alpha a_{B}\right)\right],$$

where *C* is normalization constant, $a_{B}=4\pi \varepsilon \varepsilon_{0}\hbar^{2}/\left(\mu e^{2}\right)$ is the exciton Bohr radius. Here, α is a quantum number running discrete set of values found from the boundary condition $dW_{\alpha ,1/2}\left(0\right)/dt=0$. The energy $E_{0}$ corresponds to the exciton binding in a ring of the infinite radius R→∞

(9) $$E_{0}=-\hbar^{2}/2\mu a_{B}^{2}\alpha_{0}^{2},$$

where $\alpha_{0}$ is the lowest value of quantum number α. For finite values of *R*, the energy of the internal dynamics in tight-binding approximation can be represented as:

(10) $$E_{\mathrm{int}}=\langle \chi \left(\vartheta ,R\right)|{\hat{H}}_{exc}|\chi \left(\vartheta ,R\right)\rangle =E_{0}+E_{1}+E_{2},$$

where

(11) $$\begin{array}{cc} E_{1}=\int & \left[\frac{\alpha_{C}}{R|\nu |+\gamma d}-\frac{\alpha_{C}}{2R|\sin\nu /2|+\gamma d}\right]{\left|U_{\alpha_{0}}\left(R\nu \right)\right|}^{2}Rd\nu \\ E_{2}=\int & \left[\frac{\alpha_{C}}{R|\nu |+\gamma d}-\frac{\alpha_{C}}{2R|\sin\nu /2|+\gamma d}\right]U_{\alpha_{0}}\left(R\nu \right)U_{\alpha_{0}}\left(R\left(\nu +2\pi \right)\right)Rd\nu . \end{array}$$

**Figure 2:**
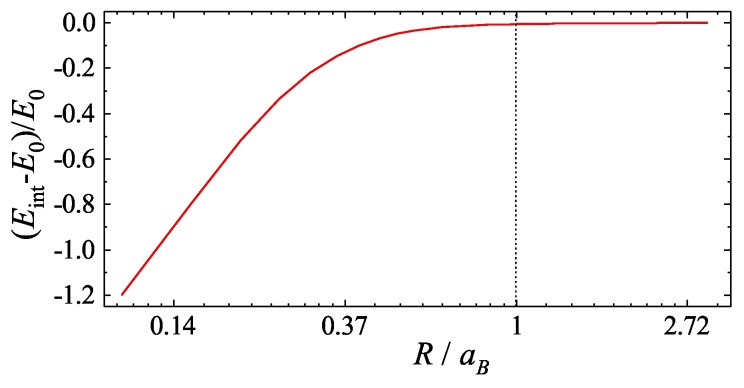
Correction to the binding energy of an exciton stemming from the finite value of the radius of the ring *R* calculated according to Equation (10). The correction plays essential role only in the region $R<a_{B}$, while for higher values of the radius the binding energy quickly converges to $E_{0}$.

We note that, regardless of the structure parameters, the second-order correction stemming from the overlap with the nearest neighbor term is negligibly small, i.e., $E_{2}\ll E_{1}$. In Figure 2, we plot the correction to the exciton energy stemming from the finite value of *R*. It is clearly seen that in the limit $R>a_{B}$ this correction becomes negligible and internal exciton state is fully determined by Coulomb interaction only. Hence, the exciton ground state wave function can be well approximated by the expression

(12) $$\chi \left(\vartheta \right)\approx CW_{\alpha_{0},1/2}\left[2\left(|R\vartheta |+\gamma d\right)/\left(\alpha a_{B}\right)\right],$$

which is used in the further calculations.

## 3. Two Exciton Energy Spectrum

We further analyze the energy spectrum of two exciton system where each of the excitons is localized in separate rings. The symmetry of the structure imposes certain restrictions on the nature of the inter-exciton Coulomb interaction, affecting the energy spectrum. It is well known that, in the case of significant overlap between excitonic wave functions, the interaction is dominated by the exchange effects [44,45,46,47]. Here, due to the strong localization of excitons inside the rings, we neglect the tunneling effects, thus accounting for the direct interaction channel between excitons only. The tunneling effects can become significant in the case of the small distance between the rings and weak radial confinement. For the barrier height of ∼500 meV [64], the highest value of radial overlap $\int rdrf_{1}^{*}\left(r\right)f_{2}\left(r\right)$ (with $f_{1}$ and $f_{2}$ being the electron radial wavefunctions in the internal and external rings, respectively) for the considered parameters is 0.1, and rapidly drops with the distance, thus allowing to discard the impact of tunneling. In the limit of the narrow rings, the corresponding interaction Hamiltonian reads:

(13) $$\begin{array}{cc} V_{\mathrm{int}}={\tilde{\alpha }}_{C} & \left[-\frac{1}{\sqrt{R_{1}^{2}+R_{2}^{2}-2R_{1}R_{2}\cos\left(\varphi_{e1}-\varphi_{h2}\right)}}\right.-\frac{1}{\sqrt{R_{1}^{2}+R_{2}^{2}-2R_{1}R_{2}\cos\left(\varphi_{h1}-\varphi_{e2}\right)}}\\ \; & +\frac{1}{\sqrt{R_{1}^{2}+R_{2}^{2}-2R_{1}R_{2}\cos\left(\varphi_{e1}-\varphi_{e2}\right)}}\left.+\frac{1}{\sqrt{R_{1}^{2}+R_{2}^{2}-2R_{1}R_{2}\cos\left(\varphi_{h1}-\varphi_{h2}\right)}}\right], \end{array}$$

where $R_{1}$ and $R_{2}$ denote the effective radii of internal and external rings, respectively. Here, ${\tilde{\alpha }}_{C}=e^{2}/\left(4\pi \varepsilon_{1}\varepsilon_{0}\right)$, with the $\varepsilon_{1}$ denoting the dielectric permittivity of surrounding media. To be specific, we consider the latter to be represented by CdSe, with the permittivity reading as $\varepsilon_{1}=10.2$ [65]. We further assume that the scattering processes do not affect the exciton internal state. In this assumption, the dynamics of two exciton system can be described by the following effective Hamiltonian:

(14) $$\begin{array}{c} \hat{H}=-\frac{\hbar^{2}}{2MR_{1}^{2}}\frac{\partial^{2}}{\partial \varphi_{1}^{2}}-\frac{\hbar^{2}}{2MR_{2}^{2}}\frac{\partial^{2}}{\partial \varphi_{2}^{2}}+\langle \chi \left(\vartheta_{1}\right)\chi \left(\vartheta_{2}\right)|V_{\mathrm{int}}\left(\xi ,\vartheta_{1},\vartheta_{2}\right)|\chi \left(\vartheta_{1}\right)\chi \left(\vartheta_{2}\right)\rangle , \end{array}$$

where $\xi =\varphi_{1}-\varphi_{2}$, and the interaction potential is averaged over the wavefunction of the internal motion of each of the excitons. In the limit of the large radii of the rings ($a_{B}\ll R_{1}$), the internal coordinate of each exciton $|\vartheta_{1(2)}|$ is a small parameter and we can use Taylor expansion for $V_{\mathrm{int}}$. The calculations shows that the lowest order non-vanishing terms are proportional to $\langle \vartheta_{1}^{2}\vartheta_{2}^{2}\rangle \approx {\left(0.4a_{B}\right)}^{4}/\left(R_{1}^{2}R_{2}^{2}\right)$, and the interaction potential gets the following form (see Appendix A):

(15) $$U_{\mathrm{int}}\left(\xi \right)\equiv \langle \chi \left(\vartheta_{1}\right)\chi \left(\vartheta_{2}\right)|V_{\mathrm{int}}\left(\xi ,\vartheta_{1},\vartheta_{2}\right)|\chi \left(\vartheta_{1}\right)\chi \left(\vartheta_{2}\right)\rangle =\frac{9{\tilde{\alpha }}_{C}{\left(m_{e}^{2}-m_{h}^{2}\right)}^{2}{\left(0.4a_{B}\right)}^{4}}{{\left(m_{e}+m_{h}\right)}^{4}}\frac{\left(7/12\right)\cos^{2}\left(\xi \right)-1/3}{{\left(R_{1}^{2}+R_{2}^{2}-2R_{1}R_{2}\cos\left(\xi \right)\right)}^{5/2}}.$$

In terms of the center of mass and relative angular coordinates of the two excitons, $\eta =(\varphi_{1}+\varphi_{2})/2$ and ξ the Hamiltonian in Equation (14) can be rewritten in the form:

(16) $$\hat{H}={\hat{H}}_{\mathrm{CM}}+{\hat{H}}_{\mathrm{rel}}+{\hat{H}}_{\mathrm{mix}},$$

where

(17) $${\hat{H}}_{\mathrm{CM}}=-\frac{\hbar^{2}}{2\left(2M\right)R_{\mathrm{eff}}^{2}}\frac{\partial^{2}}{\partial \eta^{2}},$$

(18) $${\hat{H}}_{\mathrm{rel}}=-\frac{\hbar^{2}}{2\left(M/2\right)R_{\mathrm{eff}}^{2}}\frac{\partial^{2}}{\partial \xi^{2}}+U_{\mathrm{int}}\left(\xi \right).$$

(19) $${\hat{H}}_{\mathrm{mix}}=-\frac{\hbar^{2}}{2\left(M/2\right)R_{\mathrm{eff}}^{2}}\zeta \frac{\partial^{2}}{\partial \xi \partial \eta },$$

with

(20) $$R_{\mathrm{eff}}=\frac{\sqrt{2}R_{1}R_{2}}{\sqrt{R_{1}^{2}+R_{2}^{2}}},\;\zeta =\frac{R_{2}^{2}-R_{1}^{2}}{R_{1}^{2}+R_{2}^{2}}.$$

We note that the center of mass and relative dynamics of two exciton state generally cannot be separated due to the presence of mixing term. However, in the case when both quantum rings are large enough and are close to each other, so that condition $R_{2}-R_{1}\ll R_{1},R_{2}$ is satisfied, the parameter ζ becomes negligibly small, meaning that internal and center of mass dynamics can be treated separately.

**Figure 3:**
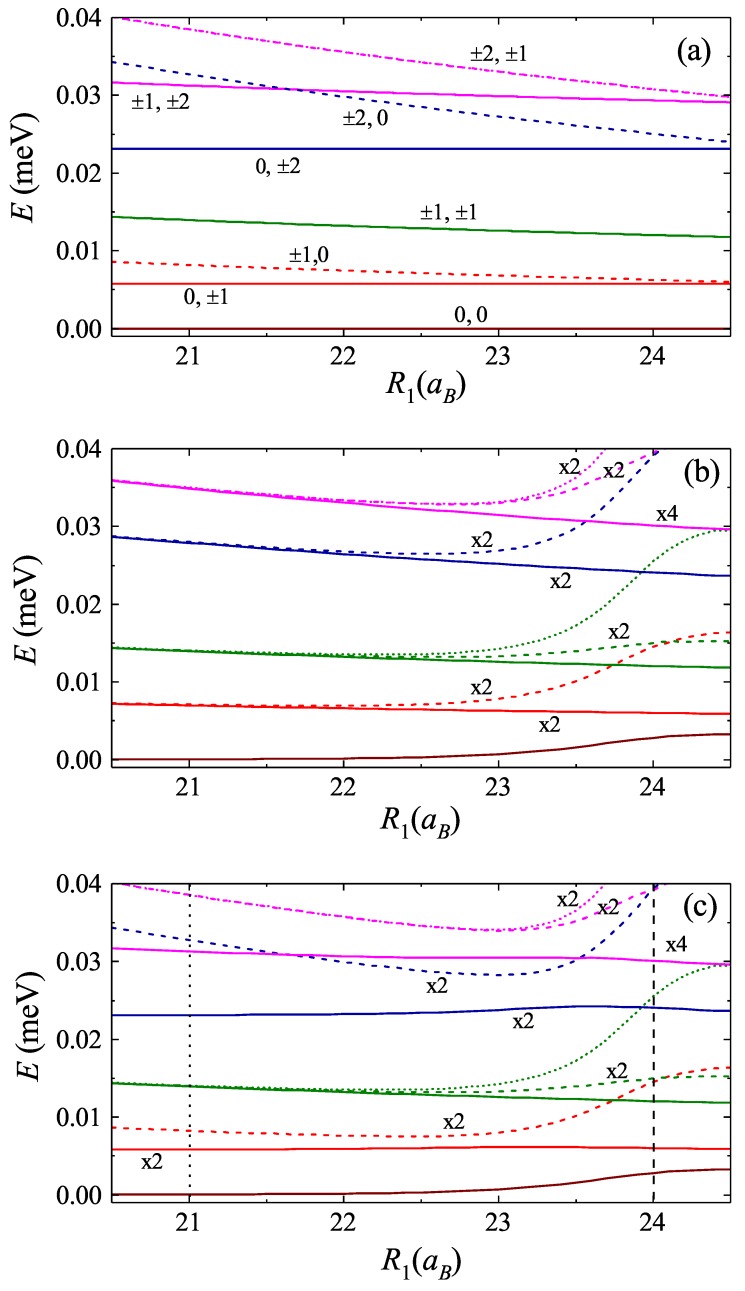
The energy spectrum of the two exciton dynamics. Here, the radius of external ring is chosen as $R_{2}=25a_{B}$: (**a**) the case of non-interacting excitons; (**b**) the mixing term is neglected; and (**c**) the full spectrum. The presence of Coulomb interactions lifts the energy degeneracy for the case of small distance between the rings (**b**,**c**), while the mixing term lifts the degeneracy in the limit of distant rings (**a**,**c**). The notation “xn” indicates the level of degeneracy. The notations in (**a**) are explained in the main text.

The energy spectrum of the Hamiltonian in Equation (16) can be found by means of exact diagonalization method. We note that here the circular symmetry of the problem imposes certain peculiarity to the structure of energy spectrum. Particularly, it is instructive to consider first the case of non-interacting excitons. Then, the problem becomes exactly solvable in the initial basis of the Hamiltonian in Equation (14). Using the cyclic boundary condition, one has the wave function of the form $\Phi_{l_{1},l_{2}}\left(\varphi_{1},\varphi_{2}\right)=Ce^{il_{1}\varphi_{1}}e^{il_{2}\varphi_{2}}$, with the corresponding energy reading as $E_{l_{1},l_{2}}=\hbar^{2}l_{1}^{2}/\left(2MR_{1}^{2}\right)+\hbar^{2}l_{2}^{2}/\left(2MR_{2}^{2}\right)$, where $l_{1,(2)}=0,\pm 1,\pm 2,\cdots$ is the angular quantum number of the exciton in the internal (external) ring. The energy spectrum as a function of ring radius $R_{1}$ and for fixed value of $R_{2}$ is shown in Figure 3a. For numerical simulations, we use the parameters of ZnO quantum ring, with electron and hole effective masses $m_{e}=0.24m_{0}$ and $m_{h}=1.21m_{0}$, respectively [66] ($m_{0}$ is free electron mass). The choice of the material is dictated by the possibility to fulfill the condition $a_{B}\ll R$, justifying the use of 1D exciton Loudon model. The indices on the lines in Figure 3a correspond to the values of quantum numbers $l_{1}$ and $l_{2}$. The extra degeneracy arising at the limit $R_{1}\to R_{2}$ between the states $\left(l_{1},0\right)$, $\left(0,l_{2}\right)$ with $|l_{1}\left|=\right|l_{2}|$ is due to the fact that we neglect the Coulomb interaction.

It is remarkable that in center of mass and relative coordinates the quantum numbers characterizing the dynamics of non-interacting excitons would take a form $L=l_{1}+l_{2}$ and $l=(l_{1}-l_{2})/2$. The latter suggests the form of ansatz for exact diagonalization:

(21) $$\Phi_{L,l}\left(\eta ,\xi \right)=\frac{1}{2\pi }\sum_{l,L}e^{iL\eta }e^{il\xi }.$$

**Figure 4:**
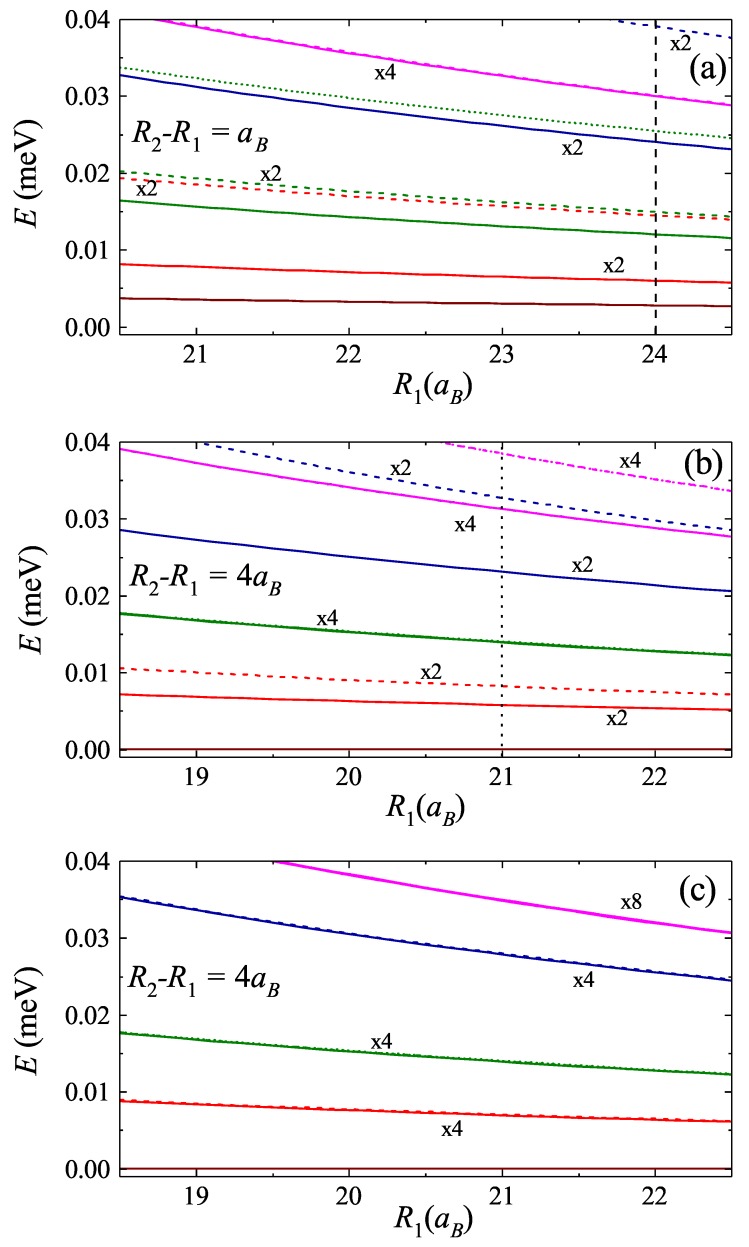
(**a**) Two exciton energy spectrum as function of ring radii for the fixed distance between the rings $R_{2}-R_{1}=a_{B}$. In the regime of small distance between the rings, the strong Coulomb interaction leads to large splitting between the corresponding pairs of states. (**b**,**c**) The energy spectrum for the ring distance $R_{2}-R_{1}=4a_{B}$ (**b**) with and (**c**) without the mixing of center of mass and relative dynamics, respectively. In the regime of large distance between the rings the strong mixing lifts the degeneracy between corresponding states. The vertical lines illustrate the equivalence with the corresponding points in Figure 3.

The results of calculation are shown in Figure 3b,c, where in Figure 3b the mixing term of the Hamiltonian in Equation (16) is neglected. The latter corresponds to the full separation of relative and center of mass dynamics. As it follows from Equation (20), in the case of small distance between the rings (large values of $R_{1}$ in Figure 3), the mixing term becomes negligibly small, resulting in the similarity of corresponding regions in Figure 3b,c. On the contrary, for the considerable difference of the radii of external and internal rings (small values of $R_{1}$ in Figure 4) the mixing becomes significant, lifting the degeneracy of exciton relative dynamics connected with the clockwise and anti-clockwise orientations of motion. Finally, the presence of Coulomb interaction lifts the energy degeneracy in the limit $R_{1}\to R_{2}$ (cf. Figure 3a). Quite remarkably, the energy splitting takes place between the states with equal angular momentum in the absence of interactions, i.e., $L=L^{\prime }$, which stems from the form of corresponding matrix element of Coulomb interaction:

(22) $$\langle \Phi_{L^{\prime },l^{\prime }}|U_{\mathrm{int}}\left(\xi \right)|\Phi_{L,l}\rangle =\delta_{L,L^{\prime }}\int e^{i(l-l^{\prime })\xi }U_{\mathrm{int}}\left(\xi \right)d\xi .$$

To examine further the contributions of the Coulomb interaction between the excitons and mixing of relative and center of mass dynamics, we study the radial dependence of energy spectrum for the fixed distance between the rings, as shown in Figure 4. Particularly, due to the small distance between the rings, Figure 4a is dominated by Coulomb interaction. On the contrary, in Figure 4b, the distance between the rings is large, leading to negligible Coulomb interaction and the strong impact of mixing term (cf. Figure 4c, where the mixing term is neglected).

Finally, we note that in the case when the quantum rings are close enough to each other so that the mixing term in the Hamiltonian in Equation (16) can be neglected, the center of mass and relative dynamics of two exciton system are separated, allowing for semi-analytic solution. Particularly, a further simplification can be done by approximating the interaction potential in Equation (18) with an effective rectangular one with the height equal to $U_{\mathrm{int}}\left(0\right)$ and the width estimated as:

(23) $$\begin{array}{c} b=\frac{1}{U_{\mathrm{int}}\left(0\right)}\int_{-\pi }^{\pi }U_{\mathrm{int}}\left(\xi \right)d\xi . \end{array}$$

In terms of the relative motion, the dynamics of the system is equivalent to the motion of a single particle in the periodic potential in Equation (18), which can be approximated by the well known Kronig–Penney model [67]:

(24) $$U_{\mathrm{rec}}\left(\xi \right)=\left\{\begin{array}{cc} \; & U_{\mathrm{int}}\left(0\right),\;2\pi n-b/2\leq \xi \leq 2\pi n+b/2,\\ \; & 0,\;2\pi n+b/2<\xi <2\pi (n+1)-b/2. \end{array}\right.$$

**Figure 5:**
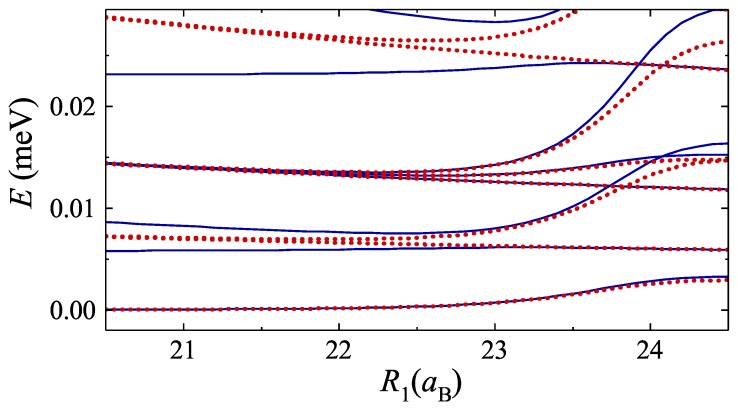
Comparison of exact (blue solid lines) and Kronig–Penney (red dots) calculation of two exciton energy spectrum. The radius of external ring is fixed as $R_{2}=25a_{B}$. In the limit of distant rings, the mixing between relative and center of mass motion becomes essential, violating the validity of approximate solution.

The energy spectrum for the relative motion of excitons can be found from the solution of the following transcendental equation:

(25) $$\frac{\beta^{2}-\kappa^{2}}{2\kappa \beta }\sinh\beta b\sin\kappa a+\cosh\beta b\cos\kappa a=\pm 1,$$

where the sign “+” (“−”) in the right hand side of Equation (25) stands for the integer (half integer) values of the *l*, $\beta =R_{\mathrm{eff}}\sqrt{M\left(U_{\mathrm{rec}}\left(0\right)-E_{\mathrm{rel}}\right)/\hbar^{2}}$, $\kappa =R_{\mathrm{eff}}\sqrt{ME_{\mathrm{rel}}/\hbar^{2}}$, a=2π−b and $E_{\mathrm{rel}}$ is the energy of the relative motion. The total energy of the system can be calculated as $E=\hbar^{2}L^{2}/2\left(2M\right)R_{\mathrm{eff}}^{2}+E_{rel}$, where it should be taken into account that the even (odd) values of *L* correspond to the integer (half integer) values of *l*. The results of calculation show an excellent correspondence with the exact calculation in the limit when center of mass and relative dynamics are well separable (see Figure 5).

## 4. Conclusions

We investigated theoretically the structure of two exciton states in double coaxial quantum rings. We characterized single exciton states in individual rings using one-dimensional Loudon model, and used the corresponding wave functions for the analysis of the properties of the Coulomb interactions between the excitons. We demonstrated the formation of specific energy spectrum of two exciton system stemming from the interplay between cyclic symmetry of the problem and the periodic Coulomb interactions. It was found that the Coulomb interactions between excitons lift the energy degeneracy for the case of small distance between the rings, while in the limit of distant rings the degeneracy is eliminated due to the mixing the center of mass and relative angular motions. Finally, we found that in the limit of small distance between the rings the energy spectrum can be described semi-analytically within the frameworks of Kronig–Penney model.

## Appendix A. The Derivation of Exciton–Exciton Coulomb Scattering Potential

The Coulomb interaction potential between two excitons (Equation (13)) can be rewritten in the form

(A1) $$\begin{array}{cc} V_{\mathrm{int}} & =\frac{e^{2}}{4\pi \varepsilon \varepsilon_{0}\sqrt{R_{1}^{2}+R_{2}^{2}}}\\ & \times \left[-\frac{1}{\sqrt{1-\rho \cos(\xi +\delta_{1})}}-\frac{1}{\sqrt{1-\rho \cos(\xi +\delta_{2})}}+\frac{1}{\sqrt{1-\rho \cos(\xi +\delta_{3})}}+\frac{1}{\sqrt{1-\rho \cos(\xi +\delta_{4})}}\right], \end{array}$$

where

(A2) $$\rho =\frac{2R_{1}R_{2}}{R_{1}^{2}+R_{2}^{2}},$$

and

(A3) $$\delta_{1}=\frac{m_{h}\vartheta_{1}+m_{e}\vartheta_{2}}{m_{e}+m_{h}},\;\delta_{2}=-\frac{m_{e}\vartheta_{1}+m_{h}\vartheta_{2}}{m_{e}+m_{h}},\;\delta_{3}=\frac{m_{h}\left(\vartheta_{1}-\vartheta_{2}\right)}{m_{e}+m_{h}},\;\delta_{4}=\frac{m_{e}\left(\vartheta_{2}-\vartheta_{1}\right)}{m_{e}+m_{h}}.$$

Applying the expansion $\cos\left(\xi +\delta_{i}\right)\approx \cos\xi \left(1-\delta_{i}^{2}/2\right)-\sin\xi \left(\delta_{i}-\delta_{i}^{3}/6\right)$, and taking into account that $\delta_{i}$ are small parameters, we yield

$$\begin{array}{cc} V_{\mathrm{int}}\approx & \frac{e^{2}}{4\pi \varepsilon \varepsilon_{0}\sqrt{R_{1}^{2}+R_{2}^{2}}\sqrt{1-\rho \cos\xi }}\\ \; & \times \left[\frac{\rho \left(\delta_{1}\sin\xi +\frac{\delta_{1}^{2}}{2}\cos\xi -\frac{\delta_{1}^{3}}{6}\sin\xi \right)}{2(1-\rho \cos\xi )}-\frac{3}{8}\frac{\rho^{2}{\left(\delta_{1}\sin\xi +\frac{\delta_{1}^{2}}{2}\cos\xi -\frac{\delta_{1}^{3}}{6}\sin\xi \right)}^{2}}{{\left(1-\rho \cos\xi \right)}^{2}}\right.\\ + & \frac{\rho \left(\delta_{2}\sin\xi +\frac{\delta_{2}^{2}}{2}\cos\xi -\frac{\delta_{2}^{3}}{6}\sin\xi \right)}{2(1-\rho \cos\xi )}-\frac{3}{8}\frac{\rho^{2}{\left(\delta_{2}\sin\xi +\frac{\delta_{2}^{2}}{2}\cos\xi -\frac{\delta_{2}^{3}}{6}\sin\xi \right)}^{2}}{{\left(1-\rho \cos\xi \right)}^{2}}\\ - & \frac{\rho \left(\delta_{3}\sin\xi +\frac{\delta_{3}^{2}}{2}\cos\xi -\frac{\delta_{3}^{3}}{6}\sin\xi \right)}{2(1-\rho \cos\xi )}+\frac{3}{8}\frac{\rho^{2}{\left(\delta_{3}\sin\xi +\frac{\delta_{3}^{2}}{2}\cos\xi -\frac{\delta_{3}^{3}}{6}\sin\xi \right)}^{2}}{{\left(1-\rho \cos\xi \right)}^{2}}\\ - & \left.\frac{\rho \left(\delta_{4}\sin\xi +\frac{\delta_{4}^{2}}{2}\cos\xi -\frac{\delta_{4}^{3}}{6}\sin\xi \right)}{2(1-\rho \cos\xi )}+\frac{3}{8}\frac{\rho^{2}{\left(\delta_{4}\sin\xi +\frac{\delta_{4}^{2}}{2}\cos\xi -\frac{\delta_{4}^{3}}{6}\sin\xi \right)}^{2}}{{\left(1-\rho \cos\xi \right)}^{2}}\right]. \end{array}$$

It is easy to check that the sum of first order terms gives zero. The sum of second order terms gives

(A4) $$V_{\mathrm{int}}^{\left(2\right)}=\frac{e^{2}}{4\pi \varepsilon \varepsilon_{0}\sqrt{R_{1}^{2}+R_{2}^{2}}\sqrt{1-\rho \cos\xi }}\frac{4\vartheta_{1}\vartheta_{2}}{1-\rho \cos\xi }\left(2\rho \cos\xi -2\rho^{2}-\rho^{2}\sin^{2}\xi \right).$$

Here, we note that $\langle \chi \left(\vartheta_{1}\right)\chi \left(\vartheta_{2}\right)|\vartheta_{1}\vartheta_{2}|\chi \left(\vartheta_{1}\right)\chi \left(\vartheta_{2}\right)\rangle =0$, thus canceling the contribution of the second order terms. All the third-order terms are odd in either $\vartheta_{1}$ or $\vartheta_{2}$, leading to the cancellation of their contribution. Finally, the sum of fourth-order terms reads as

(A5) $$V_{\mathrm{int}}^{\left(4\right)}=\frac{e^{2}}{4\pi \varepsilon \varepsilon_{0}\sqrt{R_{1}^{2}+R_{2}^{2}}\sqrt{1-\rho \cos\xi }}\frac{3\rho^{2}}{8{\left(1-\rho \cos\xi \right)}^{2}}\left(\frac{\cos^{2}\xi }{4}-\frac{\sin^{2}\xi }{3}\right)\frac{6{\left(m_{e}^{2}-m_{h}^{2}\right)}^{2}}{{\left(m_{e}+m_{h}\right)}^{4})}\vartheta_{1}^{2}\vartheta_{2}^{2}.$$

Averaging Equation (A5) over the internal dynamics of each exciton and substituting $\langle \vartheta_{i}^{2}\rangle \approx {\left(0.4a_{B}/R_{i}\right)}^{2}$, we end up with Equation (15) of the main text.
